# Trust Miscalibration Is Sometimes Necessary: An Empirical Study and a Computational Model

**DOI:** 10.3389/fpsyg.2021.690089

**Published:** 2021-08-10

**Authors:** Michael G. Collins, Ion Juvina

**Affiliations:** ASTECCA Laboratory, Department of Psychology, Wright State University, Dayton, OH, United States

**Keywords:** trust calibration, cognitive modeling, multi-arm trust game, over-reliance, under-utilization

## Abstract

The literature on trust seems to have reached a consensus that appropriately calibrated trust in humans or machines is highly desirable; miscalibrated (i.e., over- or under-) trust has been thought to only have negative consequences (i.e., over-reliance or under-utilization). While not invalidating the general idea of trust calibration, a published computational cognitive model of trust in strategic interaction predicts that some local and temporary violations of the trust calibration principle are critical for sustained success in strategic situations characterized by interdependence and uncertainty (e.g., trust game, prisoner’s dilemma, and Hawk-dove). This paper presents empirical and computational modeling work aimed at testing the predictions of under- and over-trust in an extension of the trust game, the multi-arm trust game, that captures some important characteristics of real-world interpersonal and human-machine interactions, such as the ability to choose when and with whom to interact among multiple agents. As predicted by our previous model, we found that, under conditions of increased trust necessity, participants actively reconstructed their trust-investment portfolios by discounting their trust in their previously trusted counterparts and attempting to develop trust with the counterparts that they previously distrusted. We argue that studying these exceptions of the principle of trust calibration might be critical for understanding long-term trust calibration in dynamic environments.

## Introduction and Background

Trust is generally defined as “the willingness of a party to be vulnerable to the actions of another party based on the expectation that the other will perform a particular action important to the trustor, irrespective of the ability to monitor or control that other party” (Mayer et al., 1995, p. 712) or “the attitude that an agent will help achieve an individual’s goals in a situation characterized by uncertainty and vulnerability” (Lee and See, 2004, p. 54). It is a relational construct (Hardin, 2002) based on the characteristics of both trustor and trustee as well as their interaction.

### Trust Calibration

The literature on trust seems to have reached a consensus that appropriately calibrated trust in humans or machines is highly desirable. Appropriate trust calibration is defined as a learned match between trust and trustworthiness, specifically, the case in which trustors learn to mirror the perceived dynamics of trustworthiness (Ghazizadeh et al., 2012).

#### Benefits of Trust Calibration

Kramer (1999, p. 582) argues that trust reduces transaction costs “by operating as a social decision heuristic” that replaces costly formal monitoring or measuring devices.

Relying on other agents (either human or artificial) can be a very successful strategy (e.g., Rendell et al., 2010; Ghazizadeh et al., 2012; Yaniv and Choshen-Hillel, 2012). For example, Yaniv and Choshen-Hillel (2012) found that participants who estimated uncertain quantities (the caloric value of foods) were more accurate when they had to rely on others’ opinions than when they were able to use their own judgment. These benefits are generally attributed to calibrated trust. Miscalibrated (i.e., over- or under-) trust has been thought to only have negative consequences, e.g., over-reliance and under-utilization (Muir, 1994; Parasuraman and Riley, 1997; Lee and See, 2004; Robinette et al., 2016; Okamura and Yamada, 2020). Over-reliance can lead to complacency (Parasuraman and Manzey, 2010) or gullibility (Yamagishi et al., 1999), whereas under-reliance can lead to under-utilization of potentially useful systems or agents (Lee and See, 2004); thus, both over-reliance and under-reliance can have potential detrimental effects on performance or success; in contrast, properly calibrated trust prevents over- and under-reliance and protects the trustor against error or exploitation (French et al., 2018).

#### Costs of Trust Calibration

The trustee may not always be transparent with regard to their long run trustworthiness (i.e., their true type). If the trustee is a machine, it may be impervious to the naïve trustor. Efforts to make machines more transparent may backfire as they may increase the user’s workload (Ananny and Crawford, 2018). Thus, the trustor may need to expend considerable cognitive resources to gauge the trustee’s trustworthiness and calibrate to it (Helldin, 2014). This expenditure is likely to increase as machines become more general and interact with humans over a move diverse range of environments and tasks. In general, trust reduces effort, but trust calibration requires effort.

In addition to the direct cognitive costs of trust calibration, there may be opportunity costs for trust establishment, development, or repair. Potential counterparts should be initially trusted to have an opportunity to be trustworthy or reliable. Even after a counterpart has been proven untrustworthy, it may be useful to give them another chance (or more chances) to become trustworthy. Given that trust development is a closed-loop, dynamic, and bidirectional process, trustworthiness may not be independent of trust. Conversely, when trust was established at a certain value based on old evidence of trustworthiness and more recent evidence of trustworthiness cannot be obtained, maintaining trust at the old value (i.e., keeping it calibrated) may not be the best strategy for trustors, as they may be forgoing opportunities to develop trust with other counterparts. Thus, a tight and continuous calibration may prevent opportunities to establish, develop, or repair trust with one or more counterparts.

### Predictions From General Principles of Learned Trust

While not invalidating the general idea of trust calibration, a computational cognitive model of trust in strategic interaction (Juvina et al., 2015) predicts that some local and temporary violations of the trust calibration principle are critical for sustained success in strategic situations characterized by interdependence and uncertainty (e.g., trust game, prisoner’s dilemma, and Hawk-dove). For example, players who find themselves in an equilibrium of mutual defection in iterated prisoner’s dilemma have no reason to trust their counterparts as evidence of untrustworthiness keeps accumulating. Their calibrated trust in the other player should be minimal. However, some pairs of players are able to escape this mutually destructive equilibrium and eventually reach an equilibrium of mutual cooperation. This is only possible if some players are willing to engage in costly and risky signaling, cooperate, and trust that their counterparts will reciprocate. This is a case of temporary over-trust, a violation of the trust calibration principle that, sometimes, turns out to be beneficial. The model accounts for this behavior by assessing trust necessity and temporarily adopting a learning policy that sacrifices short-term self-interest in the hopes of obtaining long-term mutual benefit.

A recent extension of the same model (Juvina et al., 2019), accounting for both human-human and human-machine interactions, predicts that trust may be discounted even in the absence of evidence of untrustworthiness if sufficient trust necessity exists. For example, if a trustor attempts to interact with a trustee in the trust game (Berg et al., 1995) and the trustee is unavailable to interact with the trustor, the model predicts a small decrement in trust, even though no evidence of untrustworthiness is observed. This is a case of under-trust, another type of violation of the trust calibration principle.

The model also explains why people with high cognitive abilities appear to over-trust their counterparts. Higher levels of cognitive ability lead to more accurate estimates of trustworthiness, which in turn lead to better trust calibration. If trustworthiness were independently distributed in the population, cognitive ability should correlate with trust calibration, but not with trust. The observed positive correlation between cognitive ability and trust (Yamagishi et al., 1999; Sturgis et al., 2010; Lyons et al., 2011) and its replication by model simulations (Juvina et al., 2019) demonstrate that trustworthiness is not independent of trust. Trustors with higher cognitive abilities were able to develop and benefit from higher levels of reciprocal trust, which in turn reinforced and maintained higher levels of trustworthiness in their counterparts.

Studying these exceptions of the principle of trust calibration might be critical for understanding long-term trust and trust calibration in dynamic environments.

### Current Work

We set out to test the predictions of under- and over-trust in an empirical study using an extension of the trust game that we called the multi-arm trust game (MATG). The following sections present the method, results, and discussion of the empirical study and the computational cognitive model that extends our previous trust models to explain the empirical results.

## Empirical Study Method

### Experimental Task

The experimental task used in this study was the MATG. The MATG is a novel experimental paradigm designed to overcome the limitations of the current experimental paradigms used in trust research (e.g., the trust game) and capture some important characteristics of real-world interpersonal and human-machine interactions, such as the ability to choose when and with whom to interact among multiple agents.

The MATG, first published by Collins^1^ in his master’s thesis, is a game of strategic interaction combining features of two commonly used games, the multi-arm bandit game (Robbins, 1952) and the trust game (Berg et al., 1995). The trust game was first used by Berg et al. (1995) to examine behavior in one-shot interactions and later used by other researchers to study behavior in repeated interactions (Dubois et al., 2012), group interactions (Ignat et al., 2019 – control condition), and over various periods of time (Strachan et al., 2020). The multi-arm bandit game (Robbins, 1952) has been used in various psychological studies to explore exploration-exploitation behavior in both animals and humans (e.g., Cohen et al., 2007). Although both the trust and the multi-arm bandit games have been used in various ways in empirical research, the combination of the two games into the MATG has three novel contributions to the literature. First, in MATG, trustors can interact with as few or as many trustees as the wish. This feature mimics the structure of real-world interactions where individuals can freely associate with others. Second, in the multi-arm bandit games, individuals must make a discrete choice to interact with a single bandit at a time, but in the MATG an individual can make both multiple choices (i.e., interact with more than one bandit at a time) and make a continuous choice deciding how much to invest in each bandit. This modification again can be seen as more ecologically valid, because individuals often have the opportunity to gauge the risk they wish to take when making a decision. Third, in the trust game, individuals often continuously interact with other people, but in the MATG interaction occurs on a more variable schedule, which again mimics more realistic human interactions. The combination of these two games provides a novel framework to examine trust behavior under more realistic and complex conditions providing a better test bed for theories of trust.

In the study presented here, the MATG is played between four players who interact repeatedly. One of the four players is randomly assigned the role of the Sender, while the other three players are assigned the role of the Receiver. Over a series of rounds in the MATG, each player makes a set of decisions depending on their role in the game. At the start of each round, both Sender and Receiver make an initial decision. First, the Sender is given a per-round endowment of 40 points and allowed to freely allocate it between themselves and the Receivers. The Sender can give as much or as little of the 40 points as they wish to either themselves or to any of the three Receivers. As the Sender allocates their per-round endowment, each Receiver must decide to interact or not with the Sender. If a Receiver decides not to interact with the Sender, then the Receiver will earn a random number of points selected from an unknown distribution. If a Receiver decides to interact with the Sender, then the Receiver will be given the number of points allocated to them by the Sender multiplied by four. For example, if a Receiver decides to interact with a Sender and the Sender allocated four points to that Receiver, then the Receiver would be given 16 points. Additionally, Receivers who choose to interact with the Sender are allowed to return any amount of their received multiplied allocation to the Sender. After all the Receivers have made their respective choices, the Sender is notified of the choices made by each of the Receivers for that round. If the Sender allocated points to a Receiver who chooses not to interact with the Sender during that round, then the Sender is notified that they could not send their points to the Receiver during this round and the Sender is given back the points allocated to the Receiver. If a Sender allocated points to a Receiver who chooses to interact with the Sender, the Sender is notified about the number of points allocated to the Receiver, the multiplied number of points that the Receiver was given, and how many points the Receiver returned to the Sender. The Sender is also told the total number of points earned during a given round. After the Sender observes the information about the Receivers, the next round begins and the same procedure is repeated.

The human participants were told that they were playing the game simultaneously with three other participants who were either humans (the animacy condition) or automated agents (the inanimacy condition). In reality, participants played with three confederate agents whose behavior was predetermined. The use of confederate agents in this study allowed us to manipulate the three trustees’ trustworthiness levels and frequency of interaction with the human participant.

### Participants

Forty-four (Age: *M* = 38.25, *SD* = 11.8, Gender: 17% female) participants were recruited from the Web site Amazon Mechanical Turk (AMT) to take part in this study and were randomly assigned to one of the two experimental conditions. Participants received a base payment of $10 for taking part in the study and earned up to an additional $10 based on their performance during the game. The average total payment was $14.48 per participant.

### Design

**Figure 1:**
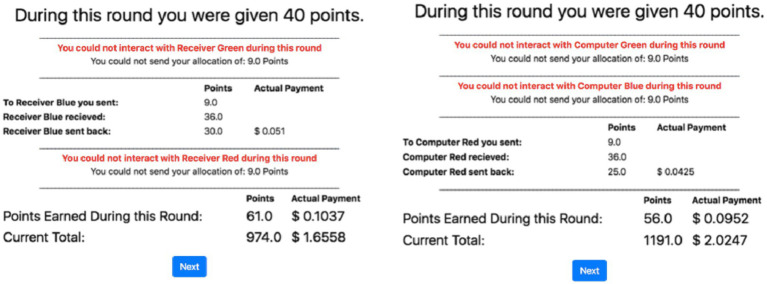
An example of the results page in the multi-arm trust game (MATG) shown to participants in the animacy condition (left plot) and the inanimacy condition (right plot).

Half of the participants were randomly allocated to an “animacy” condition, while the other half were allocated to a “inanimacy” condition. These two between-subjects conditions were formed based on the presumed identity of the participants’ counterparts, humans, or automated agents. All participants played the role of Sender with the same three confederate agents playing the role of Receiver. To help the human participants differentiate between the three confederate agents, they were color-coded and referred to as “receiver” in the animacy condition and “computer” in the inanimacy condition (Figure 1). Each experimental condition had a unique narrative regarding the identity of the confederate agents. In the animacy condition, participants were told that they are one of four participants that have been recruited to participate in the experiment. Each participant in the animacy condition was told that they have been “randomly” selected to play the role of Sender in the experiment, while the three other “participants” were assigned to play the role of Receiver. Additionally, the participants were told that the Receiver could choose not to interact with them on any round and, should they choose to do so, they could not be given their allocation during that round (Figure 1). In the inanimacy condition, the participants were told that they were interacting with three separate computer algorithms that were developed to play this game. Each time the confederate agent chose not to interact with the participant; the participant was notified in the same way as in the animacy condition.

The animacy and inanimacy conditions differed only with regard to the instructions given to the participants and the labels given to the confederate agents. The purpose of this experimental manipulation was to detect any differences between human-human and human-machine trust that may occur in this task paradigm.

Each confederate agent was randomly assigned to one of three interaction schedules, high, medium, or low. Participants were told that during each round, the confederate agents have the opportunity to choose between two tasks. The confederate agent can choose to either interact with the participant and accept the number of points sent by the participant or choose not to interact with the participant. If the confederate agent decides not to interact with the participant, they will have the opportunity to receive a reward randomly selected from an unknown distribution. If the participant decides to allocate any of their endowment to a confederate agent who has chosen not to interact with the participant during a particular round, the participant will be notified that they could not send their allocation to that counterpart during that round.

Participants were able to interact during each round with the confederate agent on the *high* interaction schedule. On the *medium* interaction schedule, the confederate agent was available to interact with the participant every three rounds (on average). On the *low* interaction schedule, the confederate agent was available to interact every six rounds (on average). Stochasticity was added to the behavior of all three confederate agents to make their availability on any given round unpredictable.

The confederate agents’ trustworthiness was also manipulated. During the first part of the game (rounds 1–70), each confederate agent returned back 75% of the multiplied number of points sent by the participant (i.e., three times the allocated amount), during rounds when it could interact with the participant. The purpose of this initially high level of trustworthiness (referred to as confederate agent’s *strategy* for simplicity) was to allow the participant to develop varying degrees of trust in the three confederate agents based on their respective interaction schedules. During the second part of the MATG (i.e., rounds 70–120), the confederate agent changed its strategy; it sent back on average the same number of points received from the participant (i.e., 25% of the multiplied amount). On average, participants did not gain or loose any points while the confederate agents used their second strategy (referred to as the neutral trustworthiness strategy). The purpose of the strategy shift from high to neutral trustworthiness was to observe whether trust would be incremented or decremented differently for different interaction schedules.

The dependent variable was the amount of points that participants allocated to each of the confederate agents, which was assumed to be a behavioral measure of trust. In addition, the participants in both animacy and inanimacy conditions answered a set of two survey measures: *trait trust*,^2^ a 24-item questionnaire including items from two different trait trust surveys from Rotter (1967) and Yamagishi (1986), and *state trust*,^3^ a custom-made 14-item questionnaire reported in Collins et al. (2016).

### Procedure

The study was reviewed and approved by the Wright State University’s Institutional Review Board. Participants were recruited from the Web site Amazon Mechanical Turk (AMT) and gave their consent to participate. Afterward, the participants read the instructions for the study and took the trait trust survey. Then, they played 120 rounds of the MATG. After completing the game, all participants took the state trust survey. After completing the experiment, the participants were debriefed to the true nature of the study and paid for both their time and performance.

### Hypotheses

Consistent with reports from the literature about observed differences between interpersonal and human-machine trust (Dzindolet et al., 2003; Lee and See, 2004; de Visser et al., 2016), we hypothesized that trust calibration and its exceptions (under- and over-trust) might manifest differently for the animacy and inanimacy conditions.

Our hypothesis regarding the differences between interaction schedules was informed by our previous cognitive model (Juvina et al., 2019) predicting that prior trust would be discounted when the current level of trust is determined based on perceived evidence of trustworthiness. In the first 70 rounds, we expect to see lower trust in the medium interaction schedule and even lower in the low interaction schedule than in the high interaction schedule. This hypothesis is incompatible with the generally accepted tenet of the trust calibration theory, according to which trust tends to mirror the dynamics of trustworthiness. The underlying trustworthiness of the three confederate agents is the same: They all return three times as many points as they receive; they only differ with regard to their interaction schedule. Thus, any observed differences between trust in the three confederate agents must be related to the different interaction schedules, specifically to the discounting of trust as a function of recency of interaction (i.e., more discounting for less recent trust), which in turn can be interpreted as an opportunity cost for delayed or infrequent interaction.

When the strategy of the confederate agents switches from high to neutral trustworthiness, the three interaction schedules are no longer associated with different opportunities for gain, as the amount returned is equal, on average, to the amount sent for all three confederate agents, resulting in a net value of zero. The participants are not better off investing in any of their counterparts than keeping their endowment to themselves. Thus, we hypothesize that trust in all three confederate agents will decrease in the last 50 rounds. Additionally, our previous model (Juvina et al., 2019) predicts that when trust is low, trust necessity increases, and trustors may adopt new trust development strategies. As the MATG was a novel experimental paradigm, we did not have clear expectations about the nature of these new strategies. Taking guidance from our previous model (Juvina et al., 2015) that was developed for different game paradigms (prisoner’s dilemma and Chicken), we hypothesized that we would see temporary violations of the trust calibration principle in the direction of over-trust.

## Empirical Study Results

### Data Cleaning

Before the data were analyzed, we checked them for quality. Inspecting online-collected data for quality is a recommended practice (Crump et al., 2013). The data obtained from some participants were different than the data of most other participants and appeared to indicate that those participants did not comply with the experimental instructions or were not sensitive to the experimental manipulations. A longitudinal k-means cluster analysis was run on the participants’ total allocation in both the animacy and inanimacy conditions. Two clusters were identified in the animacy condition and three in the inanimacy condition. Visual inspection of the plotted data in each cluster suggested that the smallest cluster in each condition was also the one containing the most erratic and less interpretable data. Thus, we excluded the smallest cluster from each condition, resulting in the exclusion of six participants from the animacy condition and five participants from the inanimacy condition.

### Main Effects and Interactions

**Figure 2:**
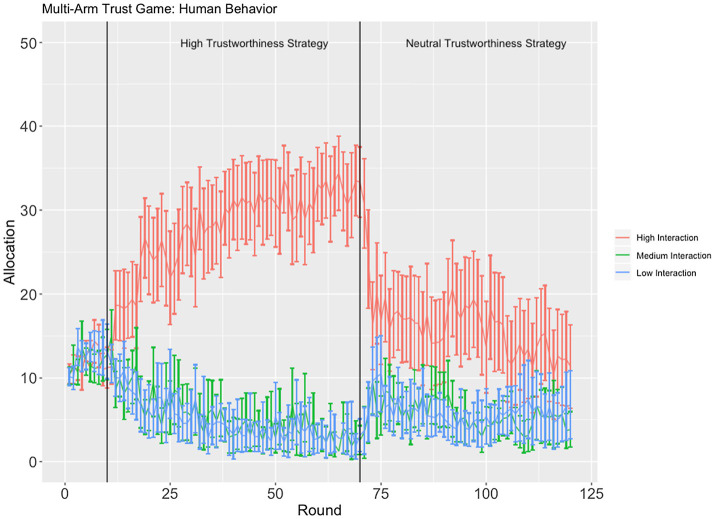
Time course of allocation (proxy for behavioral trust) as a function of interaction schedule, strategy, and round. Error bars are 95% confidence intervals.

A linear mixed-effects analysis was used, regressing the participants’ per round allocation onto round, interaction schedule (high/medium/low), strategy (high/neutral trustworthiness), and identity (in/animacy), and including a unique intercept and a slope for each participant. The full model was compared to two simpler models, intercept-only which excluded the unique random slope and a standard linear model. A model comparison based on AIC found that the full model (*AIC* = 73129.75) explained an additional amount of variance compared to both the intercept-only model (*AIC* = 75104.25) and the standard linear regression model (*AIC =* 75280.24) despite the full model’s additional complexity. According to the full model, we did not find evidence for a main effect of confederate agent identity (*p >* 0.05), an interaction between confederate agent identity and strategy (*p >* 0.05), or a four-way interaction between round, identity, strategy, and interaction schedule (*p >* 0.05). A significant three-way interaction was found between interaction schedule, strategy, and round [*F*(2, 10,030) = 308.8, *p* < 0.001, Cohen’s *f* = 0.25; Figure 2].

While the confederate agents used the high trustworthiness strategy, the participants were found to significantly increase their allocation to the confederate agent on the high interaction schedule, while decreasing their allocations to the confederate agents on the medium and low interaction schedules. We failed to find a significant difference between the allocation rates to the confederate agents on the medium and low interaction conditions (*p >* 0.05). When the confederate agents changed their strategy from high to the neutral trustworthiness, the participants were found to sharply decrease their allocation to the confederate agent on the high interaction schedule and temporarily increase their allocations to the confederates on the medium and low interaction schedules.

**Figure 3:**
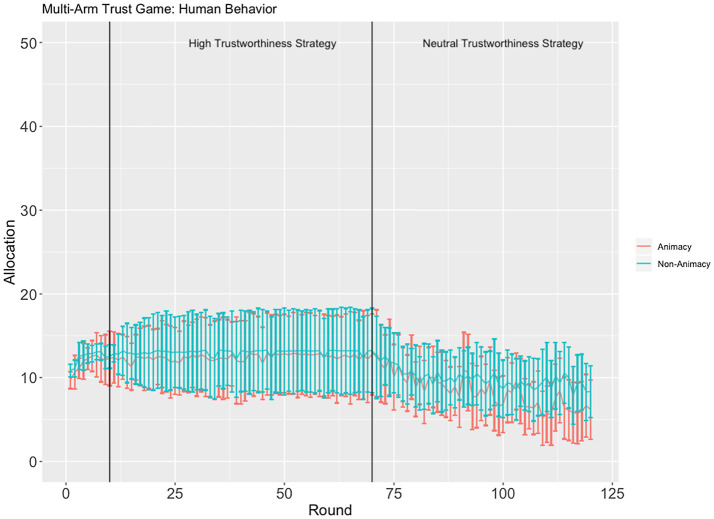
The average ±95% CI allocation across all three confederate agents in the animacy (red) and inanimacy (blue) conditions.

Another significant three-way interaction was found between identity (in/animacy), strategy, and round, *F*(1, 10,030) = 8.7327, *p* < 0.001, Cohen’s *f* =0.03 (Figure 3). This interaction appears to be entirely driven by a significant difference between the rate of decreasing allocations to confederate agents using the neutral trustworthiness strategy, *t*(1, 3.224) = 3.224, *p* < 0.006. Allocations decrease at a slightly faster rate in the animacy condition as compared to the inanimacy condition (see Figure 3). Although this finding is in line with our hypothesis that allocation behavior would manifest differently between the animacy and inanimacy conditions, the Cohen’s *f* measure for this effect was very small (*f =* 0.03), suggesting that the effect might not be robust.

Other interactions and main effects are not reported in detail here as they are consistent with the presented effects and can be unambiguously interpreted based on Figures 2, 3.

### Survey Results

A positive yet non-significant correlation between the participants’ self-reported trait trust and their total first round allocations was found*, r =* 0.33, *p =* 0.08, consistent with the intuition that participants used their trait trust to guide their initial allocation decisions when information about the trustworthiness of their counterparts was absent.

A second linear mixed-effect model regressing the participant’s state trust onto confederate agent’s identity, interaction schedule, and average allocation, with participant as random intercept was run. As expected, a main effect of interaction schedule was found, *F*(2, 48) = 13.33, *p* < 0.001, Cohen’s *f =* 0.75. Participants’ state trust in the confederate agent on the high interaction schedule was significantly higher (*M* = 3.58, *SD =* 0.85) than in the confederate agent on the medium (*M =* 2.90, *SD =* 0.85), *t*(48) = 4.113, *p* < 0.001, and low (*M* = 276, *SD* = 0.93) interaction schedules, *t*(48) = 4.78, *p* < 0.001, mirroring the effect of interaction schedule on allocations (proxy for behavioral trust).

## Discussion of Empirical Results

The animacy and inanimacy conditions were not as different as we expected. We did not find evidence for a main effect of confederate agent identity or an interaction between confederate agent identity and strategy. The only significant difference we found was a faster rate of trust reduction for the animacy condition as compared to the inanimacy condition when the confederate agents employed the neutral trustworthiness strategy. However, this result is very small and contradicts previous reports of higher trust resilience (thus slower trust decrement) in humans as compared to trust in automated systems (e.g., de Visser et al., 2016), casting doubts about its robustness.

The effect of the counterpart’s interaction schedule was overall congruent with our expectations and the predictions of our previous model regarding trust discounting (Juvina et al., 2019). In the first 70 rounds, even though the underlying trustworthiness of the three confederate agents was identical, we observed lower behavioral trust in the medium and low interaction schedules than in the high interaction schedule consistent with the idea that prior trust is discounted as a function of recency of interaction. In other words, delayed or infrequent interaction could cause future discounting of trust even if trustworthiness remains at a high level. We also hypothesized a significant difference between allocations to confederate agents on the medium or low interaction schedules, which was not found. Possible explanations for the similar allocations to confederate agents on the medium and low interaction schedules are a floor effect and a winner-takes-all maximizing strategy.

**Figure 4:**
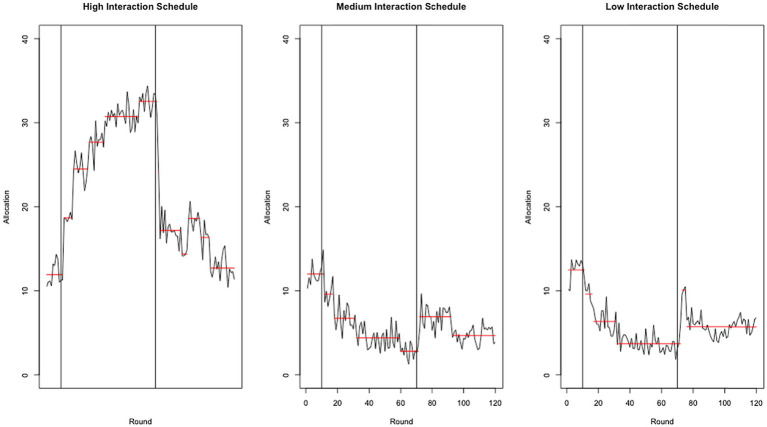
Average amount sent (allocation) by participants by round, interaction schedule, and strategy. The red lines mark the change points detected by the PELT method (Killick et al., 2012).

When the strategy of the confederate agents switched from high to neutral trustworthiness (round 70, marked with a vertical line in Figure 4), the participants sharply decreased their allocations to (i.e., trust in) their counterpart on the high interaction schedule (see Figure 4, left panel) and temporarily increased allocations to their least trusted counterparts (see Figure 4, middle and right panels). We see this paradoxical effect (i.e., decreasing trust in the most trusted counterpart while increasing trust in the least trusted counterparts^4^) as evidence of a trust development strategy. As predicted by our previous model (Juvina et al., 2015), under conditions of increased trust necessity, participants attempted to actively reconstruct their trust investment portfolios. Due to the setup of this study, those attempts to develop trust were never reciprocated as the counterparts maintained their neutral trustworthiness strategy. However, this trust development strategy would arguably work in the real world where trust relationships are likely to be reciprocal and a trustee would likely understand the trustor’s intent to rebuild the trust relationship. At the least, it was shown to work in other games by overcoming mutual distrust in prisoner’s dilemma and Chicken (see Juvina et al., 2015 for details).

A through inspection of Figure 4 reveals that the change in allocations after round 70 (when trustworthiness shifts from high to neutral) appears to take place almost immediately (within three rounds) for all three confederate agents, even though the low-frequency interaction agent only interacts every six rounds and the medium-frequency agent every three rounds. If the recalibration of trust were solely a function of a trustee’s return, one would expect a delay in the change for the low and medium agents. Interestingly, that delay did not occur, which implies that behavioral trust in an agent is based on more than the direct interactions with that agent. Apparently, the trustor took into consideration the behavior of the high-frequency agent to determine their trust in the medium- and low-frequency agents, which is consistent with our trust development driven by trust necessity account (see Juvina et al., 2019 for a more extensive discussion and validation of this account).

## A Computational Cognitive Model of Matg

### Model Description

A computational cognitive model for MATG extending our previous model (Juvina et al., 2019) was developed in the ACT-R cognitive architecture (Anderson, 2007). To model human behavior in the MATG, the ACT-R model uses the declarative and procedural memories and a trust-learning mechanism to anticipate the confederate agents’ behavior, develop, and extend trust (i.e., make allocations). The model learns about the behavior of the confederate agent by using instance-based learning (Gonzalez et al., 2003) and sequence learning. Over the course of the game, the model stores chunks in declarative memory, which are representations of instances that occurred during the game. Storing and recalling these previous instances of behavior allow the model to inform its decisions. Retrieval of prior instances from declarative memory is a function of the recency and frequency of their occurrence in the game. Procedural memory is used to make decisions about how to allocate the model’s endowment across the confederate agents. In ACT-R, procedural memory is reinforced by rewards from the environment. The prior history of rewards is represented as utility and affects the probability of choosing a particular action in the future.

At the start of each round, the model sequentially allocates points to one of the three confederate agents at a time, until a decision about how to allocate its endowment across the three confederate agents has been made. Which confederate agent the model allocates its endowment to first is initially random, but over time is a function of the payoff that the model receives while interacting with the confederate agent. To decide how many points to allocate to a counterpart, the model uses IBL and sequence learning to predict how many points that counterpart is likely to return. The prediction is based on the current context and all previous instances of interactions with that particular confederate agent aggregated by blending (Lebiere, 1999). After the model makes a prediction, it decides to increase or decrease its previous allocation to that counterpart depending on the predicted return and the previous rewards that the model received for increasing or decreasing allocations in contexts similar to the current one. This process is then repeated for each of the three confederate agents.

After the model makes a decision to allocate its 40 points across the three confederate agents and itself, it receives feedback about the number of points returned by each confederate agent. It is this feedback information that is used to learn about each of the confederate agents. Furthermore, the model receives a reward from its interaction with each of the confederate agents based on its trust state and behavior of the confederate agent. The model’s trust state is defined by two accumulators: trust and trust-invest, indexing state trust and trust necessity, respectively (see Juvina et al., 2015, 2019 for details). If the model’s trust accumulator is above a threshold (i.e., a state of trust), then the model receives a reward based on the joint payoff of both trustor and trustee. If the model’s trust accumulator is below threshold and its trust-invest accumulator is below threshold (i.e., a state of distrust), then the model receives a negative reward for allocating points to that confederate agent. If the model’s trust is below threshold, but the trust-invest accumulator is above threshold (i.e., a state of trust necessity), then the model receives positive reward based on the confederate agent’s payoff. In addition to the reward the model receives from each of the confederate agents, the model also receives a reward for its endowment kept (i.e., not allocated to any of the confederate agents). The sum total of these rewards is received at the end of each round, updating all the rules that fired in the previous round.

### Modeling Results and Discussion

The ACT-R model was fit to the average allocations to the three confederate agents. The following parameters were varied to fit the data: the trust and trust-invest accumulators, the trust discounting parameter, the ACT-R’s procedural learning rate, and a noise parameter. The ACT-R model’s fit was evaluated using standard measures of fit correlation (r) and root-mean-squared deviation (RMSD). In addition, the full ACT-R model was compared against “lesioned” models lacking the trust and trust-invest accumulators. The comparison of the full trust model to these partial models allowed for an evaluation of which components of the full trust model contributed to the fit. Each of the three ACT-R models was assessed by evaluating the *r* and RMSD between the average allocation to each of the three confederate agents, over the course of the entire experiment (rounds 1–120), while the confederate agents used the high trustworthiness strategy (rounds 1–70) and the neutral trustworthiness strategy (rounds 71–120; see Table 1).

**Table 1:** The correlation (*r*) and root-mean-squared deviation (RMSD) of the three models fit to the full experiment, high trustworthiness strategy (rounds 1–70), and neutral trustworthiness strategy (rounds 71–120).

	Full experiment		High trustworthiness strategy (1–70)		Neutral trustworthiness (71–120)	
	*r*	RMSD	*r*	RMSD	*r*	RMSD
Full trust model	0.92	0.14	0.96	0.51	0.83	1.05
Trust model	0.89	1.01	0.97	0.79	0.61	1.32
No trust model	0.85	1.41	0.97	0.70	0.41	4.37

**Figure 5:**
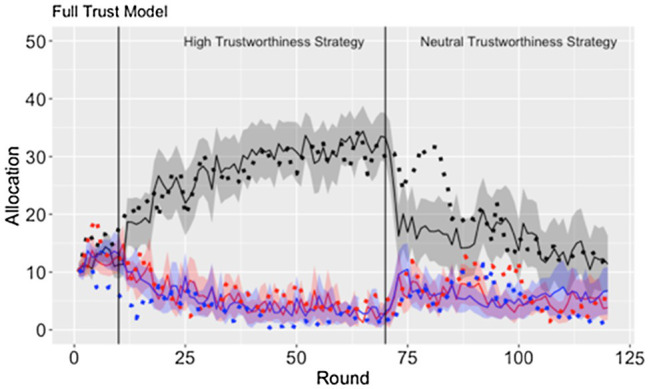
The model fit (dotted line) to the participants’ average allocation ±95% CI (solid line and ribbon) to the confederate agents on the high (black), medium (red), and low (blue) interaction schedules.

Overall, the full trust model best fits the data (Figure 5). While the confederate agents used the high trustworthiness strategy, similar fits were observed across each of the three models (Table 1). However, during the trustworthiness-neutral portion of the study, the full trust model maintained the best fit while the lesioned models decreasing their fit significantly. These findings suggest that both trust and trust-invest accumulators are important for capturing the unique trends in the participant’s behavior over the course of the experiment.

**Figure 6:**
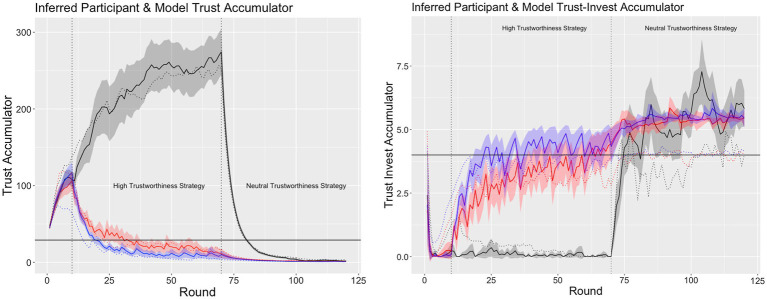
The average ±95% CI inferred trust (left plot) and trust-invest (right plot) in the high (solid black line), medium (solid redline), and low (solid blue line) interaction condition and the average model trust and trust-invest values for high (dashed black line), medium (dashed red line), and low (dashed blue line) interaction schedule.

We used the model to infer the participants’ trust and trust-invest accumulators by feeding the model the decisions that the participants made and their payoffs instead of its own decisions and payoffs. A comparison between the participants’ average inferred trust and trust-invest values and the model’s trust and trust-invest accumulators revealed a high degree of similarity [trust accumulator: *r*(358) = 0.99, *p* < 0.01; trust invest accumulator: *r*(358) = 0.91, *p* < 0.01; see Figure 6]. These results suggest that participants’ behavior can be explained from the perspective of the trust mechanisms used in the ACT-R model.

## General Discussion and Conclusion

The presented empirical study and modeling work suggest that trust calibration is a more complex process than previously assumed, particularly in complex, dynamic, and closed-loop interactions. Arguably, the MATG paradigm affords new opportunities for researchers to study trust in more realistic settings characterized by selective, non-continual interaction, and interdependence among multiple trustors and trustees. Using the MATG paradigm allowed us to empirically establish that, under conditions of increased trust necessity, participants actively reconstructed their trust investment portfolios by discounting their trust in their previously trusted counterparts and attempting to develop trust with the counterparts that they previously distrusted. Arguably, temporary and local violations of the general trust calibration principle are important for trust establishment, development, or repair. Potential counterparts should be initially trusted to have an opportunity to be trustworthy or reliable. Even after a counterpart has been proven untrustworthy, it may be useful to give them another chance (or more chances) to become trustworthy. Given that trust development is a closed-loop, dynamic, and bidirectional process, trustworthiness may not be independent of trust. Conversely, when trust was established at a certain value based on old evidence of trustworthiness and more recent evidence of trustworthiness cannot be obtained, maintaining trust at the old value (i.e., keeping it calibrated) may not be the best strategy for trustors, as they may be forgoing opportunities to develop trust with other counterparts. Thus, a tight and continuous calibration may prevent opportunities to establish, develop, or repair trust with one or more counterparts. Studying these temporary exceptions of the principle of trust calibration might be critical for understanding long-term trust development and calibration in dynamic environments.

A word of caution with regard to how widely these empirical findings can be generalized is necessary here. Two of the independent variables (trustworthiness and frequency of interaction) were treated as nominal variables even though they could take on many possible values. The cutoff points for the two variables (75%/25% and 1/3/6 trials, respectively) were selected based on the results of a pilot study (see Footnote 1, for details). It is possible that the pattern of results reported here may not generalize to different cutoff points for these variables.

The observed empirical effects were explained with the aid of a computational cognitive model developed within a cognitive architecture. The model’s explanatory mechanisms (i.e., trust discounting and strategy shift triggered by trust necessity) were derived from a series of previous models of trust in strategic interaction (Juvina et al., 2015, 2019), which aligns the current study with other efforts to develop a comprehensive theory of trust in interpersonal and human-machine interactions. In addition, the cognitive model provides a path forward for further empirical work. The model fit to the human data from this study can be used to generate predictions for other experiments using the MATG. We hope that the proposed model will be used in studies of realistic interactions (e.g., virtual teams) to understand how humans develop, maintain, and repair interpersonal or human-machine trust relationships within groups.

## Data Availability Statement

The raw data supporting the conclusions of this article will be made available by the authors, without undue reservation.

## Ethics Statement

The studies involving human participants were reviewed and approved by the Wright State University’s Institutional Review Board. The patients/participants provided their written informed consent to participate in this study.

## Author Contributions

MGC and IJ contributed equally to designing the study and writing the manuscript. MGC collected the empirical data, developed the ACT-R model, and analyzed the data. All authors contributed to the article and approved the submitted version.

## Conflict of Interest

The authors declare that the research was conducted in the absence of any commercial or financial relationships that could be construed as a potential conflict of interest.

## Publisher’s Note

All claims expressed in this article are solely those of the authors and do not necessarily represent those of their affiliated organizations, or those of the publisher, the editors and the reviewers. Any product that may be evaluated in this article, or claim that may be made by its manufacturer, is not guaranteed or endorsed by the publisher.

## Footnotes

^1^Collins, M. (2020). Trust discounting in the multi-arm trust game. unpublished master’s thesis. Dayton, Ohio: Wright State University. Available at: https://corescholar.libraries.wright.edu/etd_all/2377/

^2^An example item in the trait trust survey is “Most people are basically honest.”

^3^An example item in the state trust survey is “Receiver Red can be trusted.”

^4^The slight trust increase in the least-trusted counterparts (see Figure 4, middle and right panels) caused by a shift from high to neutral trustworthiness was not detected by the linear mixed-effects analysis, likely because the change was relatively small and brief.
